# Mobile App Delivery of the EORTC QLQ-C30 Questionnaire to Assess Health-Related Quality of Life in Oncological Patients: Usability Study

**DOI:** 10.2196/mhealth.9486

**Published:** 2018-02-20

**Authors:** Kerstin A Kessel, Marco ME Vogel, Anna Alles, Sophie Dobiasch, Hanna Fischer, Stephanie E Combs

**Affiliations:** ^1^ Department of Radiation Oncology Technical University of Munich Munich Germany; ^2^ Institute for Innovative Radiotherapy Helmholtz Zentrum München Neuherberg Germany

**Keywords:** radiation oncology, healthcare surveys, mobile applications, mobile apps, telemedicine, health-related quality of life, questionnaires, oncology

## Abstract

**Background:**

Mobile apps are evolving in the medical field. However, ongoing discussions have questioned whether such apps are really valuable and whether patients will accept their use in day-to-day clinical life. Therefore, we initiated a usability study in our department.

**Objective:**

We present our results of the first app prototype and patient testing of health-related quality of life (HRQoL) assessment in oncological patients.

**Methods:**

We developed an app prototype for the iOS operating system within eight months in three phases: conception, initial development, and pilot testing. For the HRQoL assessment, we chose to implement only the European Organization for Research and Treatment of Cancer (EORTC) Quality of Life Questionnaire-Core 30 (QLQ-C30; German version 3). Usability testing was conducted for three months. Participation was voluntary and pseudonymized. After completion of the QLQ-C30 questionnaire using iPads provided by our department, we performed a short survey with 10 questions. This survey inquired about patients’ opinions regarding general aspects, including technical advances in medicine, mobile and app assistance during cancer treatment, and the app-specific functions (eg, interface and navigation).

**Results:**

After logging into the app, the user can choose between starting a questionnaire, reviewing answers (administrators only), and logging out. The questionnaire is displayed with the same information, questions, and answers as on the original QLQ-C30 sheet. No alterations in wording were made. Usability was tested with 81 patients; median age was 55 years. The median time for completing the HRQoL questionnaire on the iPad was 4.0 minutes. Of all participants, 84% (68/81) owned a mobile device. Similarly, 84% (68/81) of participants would prefer a mobile version of the HRQoL questionnaire instead of a paper-based version. Using the app in daily life during and after cancer treatment would be supported by 83% (67/81) of participants. In the prototype version of the app, data were stored on the device; in the future, 79% (64/81) of the patients would agree to transfer data via the Internet.

**Conclusions:**

Our usability test showed good results regarding attractiveness, operability, and understandability. Moreover, our results demonstrate a high overall acceptance of mobile apps and telemedicine in oncology. The HRQoL assessment via the app was accepted thoroughly by patients, and individuals are keen to use it in clinical routines, while data privacy and security must be ensured.

## Introduction

Since the first smartphone was introduced by IBM in 1995 [1], the development of cell phones and mobile apps has been world-changing. The success of medical and health apps (labeled under mobile health [mHealth] or electronic health [eHealth]) is undeniable, with 165,000 programs in the respective leading app stores (Apple and Google) [2]. Apps for registering heart rate, blood pressure, and blood glucose are forthcoming, and apps for depression, body weight reduction, and diabetes are widely accepted. However, most of the apps are not scientifically validated [3-5]. To date, only a few apps in an oncological context exist, which allow for quality of life (QoL) assessment.

An essential key to everybody’s well-being is QoL, which the World Health Organization defined in 1946 as an, “individual's perception of their position in life in the context of the culture and value systems in which they live and in relation to their goals, expectations, standards and concerns” [6]. This broad definition includes a holistic approach to QoL, which is rarely used in medical research. In the 1980s the concept of health-related quality of life (HRQoL) evolved [7]. HRQoL is a critical criterion in the therapeutic decision-making process undertaken by health care professionals (HCPs) who are torn between the patients’ well-being, outcome, and economic considerations. Especially in oncology, where treatment is often only life-prolonging and not curative, HRQoL assessment is crucial to identify therapeutic benefit and need. The European Organization for Research and Treatment of Cancer (EORTC) developed a wide range of standardized and validated questionnaires to assess HRQoL in oncological patients. In this study we used the EORTC Quality of Life Questionnaire-Core 30 (QLQ-C30) [8], which is the core questionnaire that can be extended by cancer-specific modules.

In earlier series, we asked HCPs [9] and patients [10] about their attitudes regarding telemedicine and mobile apps in oncology. A total of 84.3% of HCPs supported the idea of an oncological app, and 97.8% found the HRQoL assessment very useful or useful. Approximately half of the patients asked were willing to use an oncological app; 75.3% of those patients were keen to send HRQoL data. In general, younger patients were more in favor of such an app (*P*=.032, r=-0.12).

Our previous results show an existing demand for oncological apps. Hence, we developed an app for HRQoL assessment in oncological patients. This work aims to present our results of the first prototype, the design/development, and patient testing.

## Methods

For the first version of the app, we decided to develop the prototype for the iOS operating system (version 8 and later) optimized for iPads (generation 2 and later), but the app also worked on iPhones (generation 4s and later). Further operating systems and device options were scheduled after the first patient testing and validation results were obtained. The development was completed within eight months in three phases: conception, initial development, and pilot testing.

### App Design and Development

Considering our previously published work on app use during cancer treatment [9-11], we specified primary functional requirements for the prototype listed in Table 1 with the following four main characteristics:

1. Clear interface and user-friendly design
2. The prototype should implement only the EORTC QLQ-C30 questionnaire (German version 3; Multimedia Appendix 1 [German], Multimedia Appendix 2 [English])
3. Data is locally stored on the device; no Internet connection or online data transfer were intended in this version
4. Login information (user identification [ID] and password) will be generated and provided by the department; this method ensured pseudonymization

The app was developed using Xcode 7.0 and Swift 2.2.

**Table 1:** Functional requirements for the prototype app.

Requirement	Details
User management	A user can register herself/himself with a given user ID and password on a device; after that, login and logout are possible on that specific device
Administration	The administrator can delete registered users on the device
	The administrator can review completed questionnaires and delete them
Questionnaire	A questionnaire can be filled out multiple times
	A questionnaire can be canceled at any time; results are not saved
	Questions can be skipped
	Answers can be changed
	Completed questionnaires can be saved (submitted) to store the results on the device
Data management	Questionnaires are stored with the information: user ID, date, given answers

### Validation

As an empirical usability evaluation method, we used a questionnaire. The survey was conducted for three months at the Department of Radiation Oncology, Klinikum rechts der Isar, Munich, Germany. Participation was voluntary and pseudonymized. Inclusion criteria for participation were: age older than 18 years, German-speaking, and being physically and mentally able to fill out a structured questionnaire on a mobile device. Research assistants supervised the patients during app use and while completing the questionnaire. The Ethics Committee of the Technical University of Munich approved the nature and content of the study with the project number 321/16 S.

After completion of the QLQ-C30 questionnaire using iPads provided by our department, we conducted the short usability survey with 10 questions (Multimedia Appendix 3). This survey inquired about patients’ opinions regarding general aspects, including technical advances in medicine, mobile and app assistance during cancer treatment, and the app-specific functions (eg, attractiveness, operability, and understandability). Statistical calculations were performed using SPSS Statistics v23 (IBM, Armonk, NY, USA) in a primarily descriptive way.

## Results

### App Design and Development

**Figure 1:**
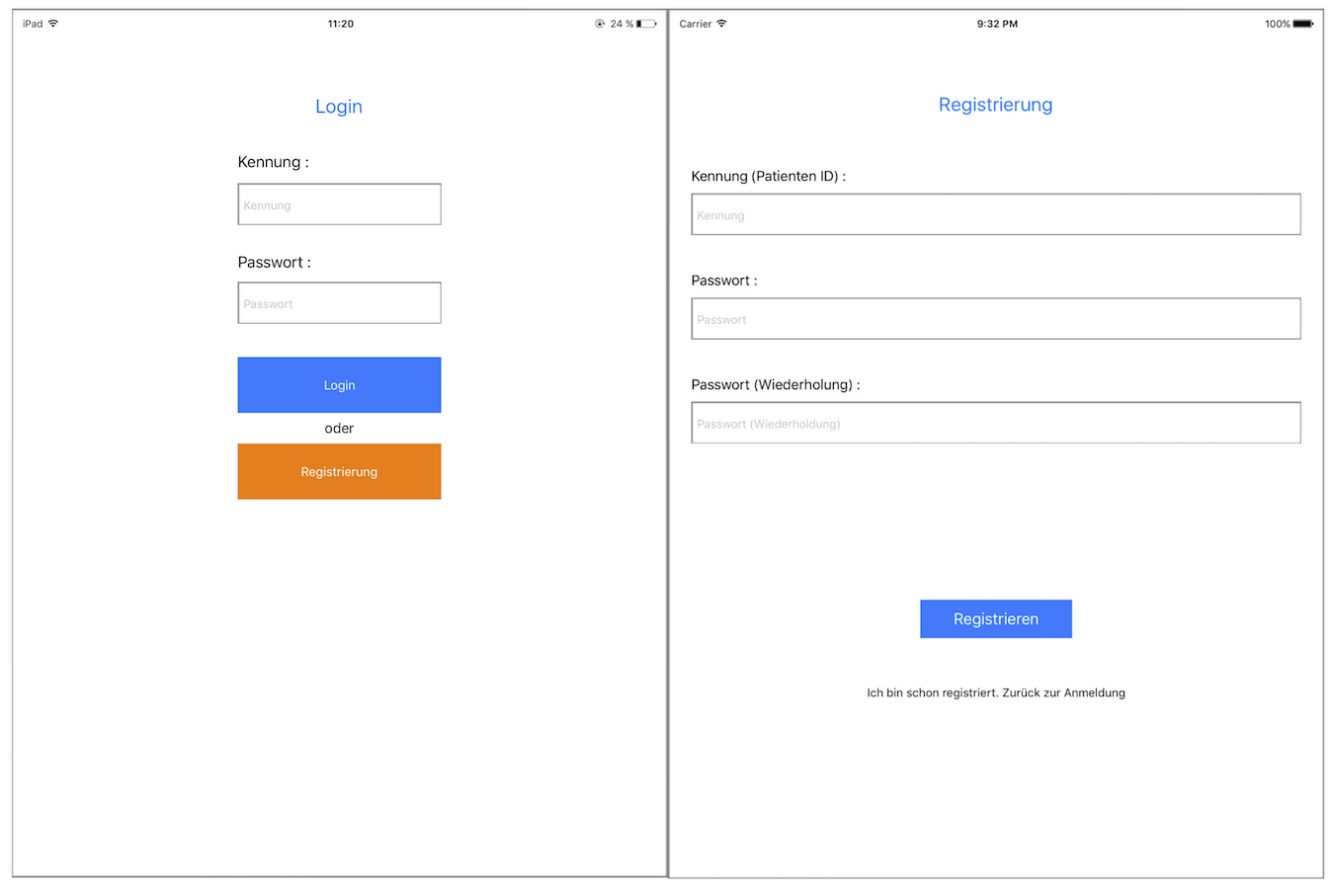
Screenshot of the login (left) and registration (right) page.

**Figure 2:**
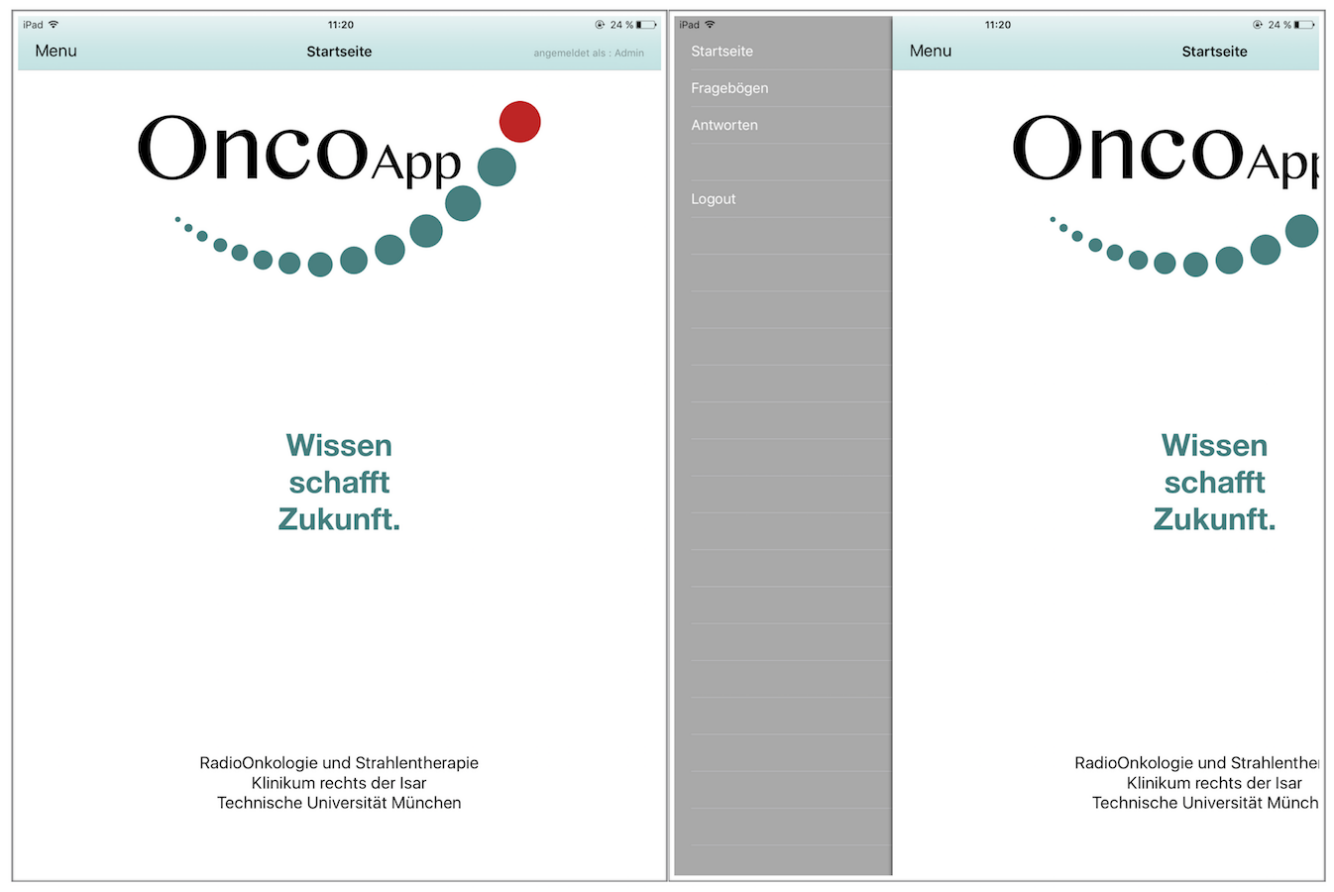
Screenshot of the start page (left) and slide bar of the menu (right). Administrator view is displayed.

After launching the app, the login page appears (Figure 1, left). If accessing the app for first time, a user ID and password need to be registered on the device (Figure 1, right). After login, the start page appears (Figure 2, left). We decided to use a slide bar for the menu, as it is easily extendable for future additional functions of the app. Users can click the button in the top left corner or swipe left on the screen to open the menu. For the prototype, the functions for starting a questionnaire, reviewing answers (administrator only), and logging out were implemented (Figure 2, right).

**Figure 3:**
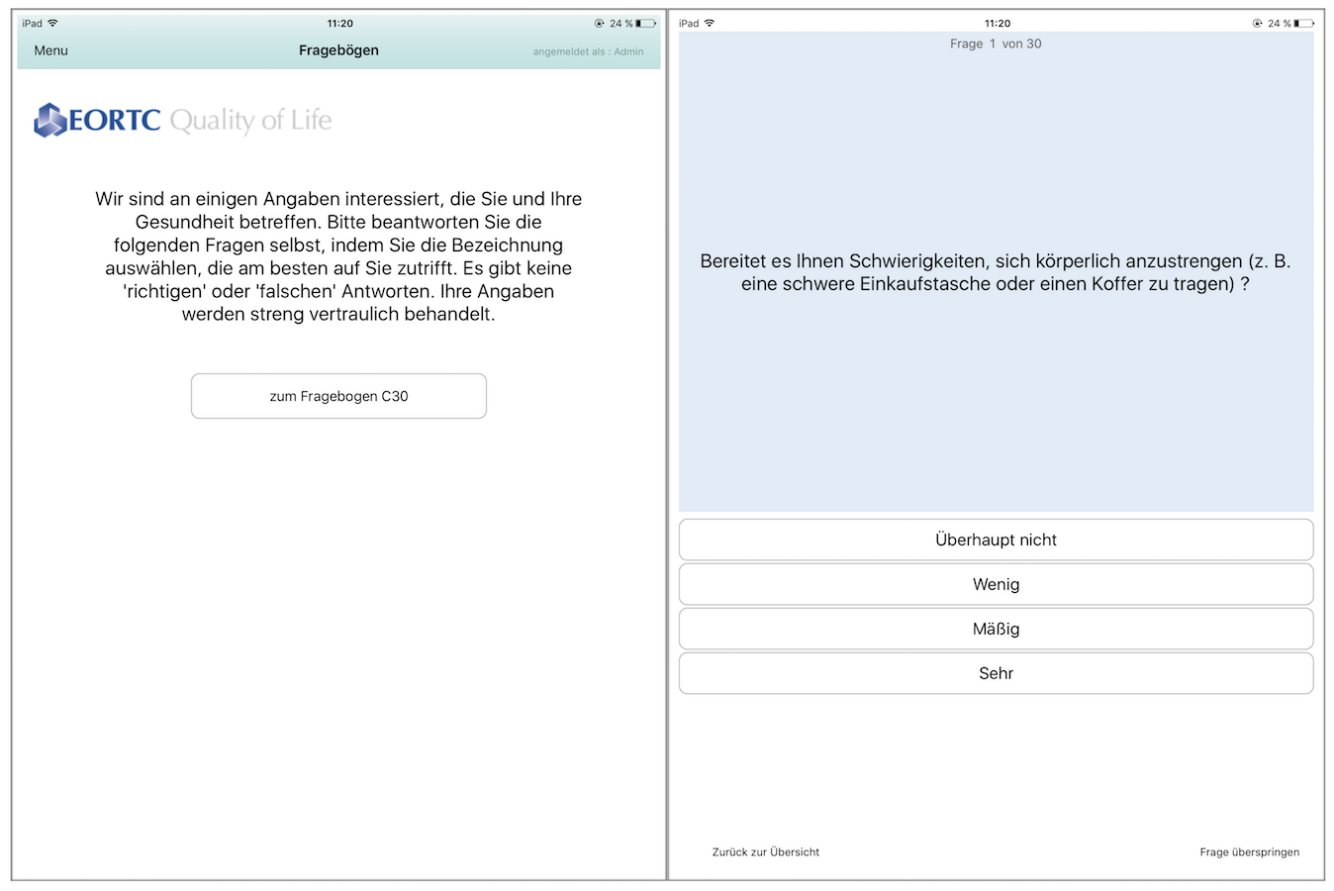
Screenshot of the start page of the European Organization for Researchand Treatment of Cancer Quality of Life Questionnaire-Core 30 (EORTC QLQ-C30; left) and the first question (right).

Figure 3 (left) shows the start page of the EORTC QLQ-C30 questionnaire. We displayed the same information that is written on the paper-based version of the questionnaire. Likewise, all 30 questions were copied from the original QLQ-C30 sheet. No alterations in wording or answers were made. Figure 3 (right) shows the first question. On the bottom of each page, the user can go back to the previous question or skip the current question. After the last question, the user is asked to save the completed questionnaire. These answers can be reviewed by the administrator (Multimedia Appendix 4).

**Figure 4:**
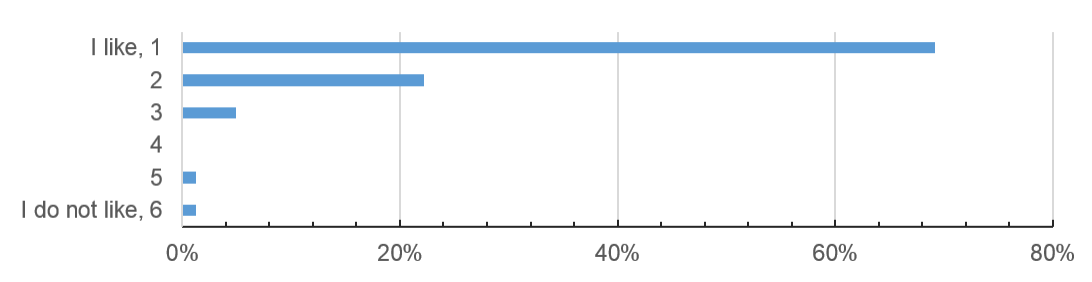
Patient opinion about mobile technologies in medicine (scale from 1 = “I like the development” to 6 = “I don’t like the development”).

**Figure 5:**
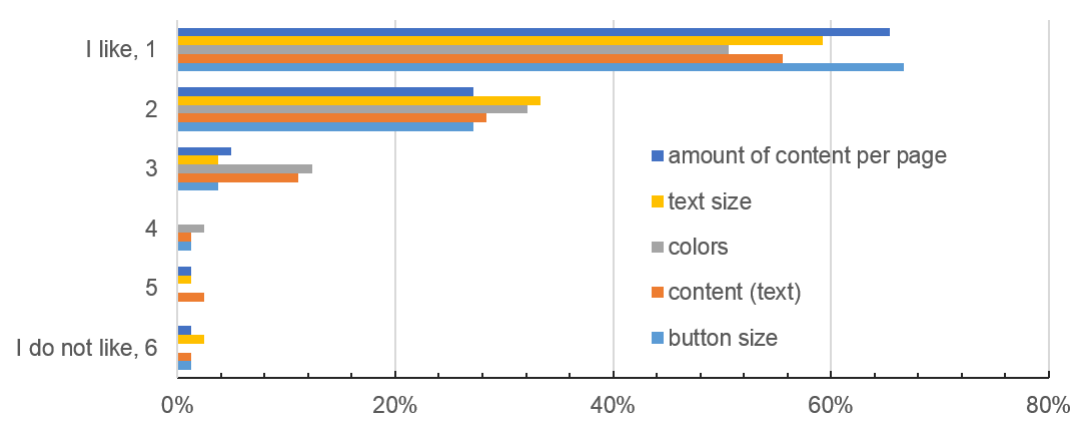
Patient opinion about the app design in terms of color, text and button size, content, and the amount of content per page (scale from 1 = “I like it” to 6 = “I don’t like it”).

### Validation

The usability of the app was tested with 81 patients (44 male, 37 female); median age was 55 years (range 21-80 years). When using the app, older patients (>60 years old) distinguished themselves from younger patients (<60 years old). The research assistants observed that while the elderly users had to ask questions about where to click and swipe on the screen, the younger users intuitively knew how to navigate through the app.

The median time for completing the HRQoL questionnaire on the iPad was 4.0 minutes (range 1.5-8.0 minutes). Of all participants, 84% (68/81) owned a mobile device. Similarly, 84% (68/81) would prefer the mobile version of the HRQoL questionnaire instead of the paper-based version that they usually get during a clinical visit. Using the app in daily life during and after cancer treatment would be supported by 83% (67/81) of participants, which corresponds with their general opinion about mobile technologies in medicine (described in Figure 4). Patients were satisfied with the current development and the introduction of the app into clinical life. In the prototype version of the app, data were stored on the device; in the future, 79% (64/81) of the patients would agree to transfer data via the Internet.

The operability and navigation of the app were rated as intuitive by 95% (77/81) and 93% (75/81) of participants, respectively. Six participants stated opportunities for improvements regarding the HRQoL questions. However, these cannot be changed as they follow a standardized structure and wording that has been established by the EORTC. Regarding the attractiveness, two patients wished for a setting option to change the font size; five had difficulties finding the menu at first. Figure 5 shows the patients’ opinions on the app interface and design.

## Discussion

The long-term aim of app-assisted care is to generate higher treatment quality and HRQoL for our patients. In this study, we present our first app-prototype for regular HRQoL assessment, along with the patient usability testing. Most of the participants (84%, 68/81) owned a mobile device and therefore were appropriately equipped to use an app. Similarly, 84% (68/81) of participants would prefer a mobile version of HRQoL assessment instead of a paper-based version, while 83% (67/81) would use it in daily life. Such an app would increase clinical efficiency by reducing paperwork and costs, and enhance patient empowerment. Our results show that mobile technologies are widely accepted; 96% (78/81) of the patients scale their opinion about telemedicine as *positive* (scale 1-3; Figure 4).

The users in this study were cancer patients, and the concept of the prototype was to implement a clean and simple app, with the primary goal to be user-friendly. The oncological patient population includes individuals of young and old age. While implementing apps in clinical routine, age and technical skills always seem to be a problem. In our study, patients >60 years old needed more assistance than younger patients. However, Smith et al showed a growing trend of smartphone use amongst elderly people (>65 years) in the United States, with 18% in 2014 [12] and 27% in 2015 [13]. Older individuals incrementally adapt to mobile technology. Nevertheless, it is essential to develop apps for older patients with a focus on operability and understandability. Intensive preuse teaching might be necessary in some cases.

Although we recently showed a higher usage of Android phones (52.9%) than iOS phones (37.2%) in our patient population [10], we rationally decided to develop the first version of the app for iPhones and iPads. iOS development is cheaper [14] and more comfortable, as apps only need to meet the criteria of two model types. However, a version of our app for Android is planned in the near future.

The interface was designed in light, cool colors and we omitted any animation or gamification. Overall, we used hues of the blue and green family, which tend to psychosocially calm down the already anxious cancer patients [15]. We chose to use dark characters on white background. Piepenbrock et al [16] showed an advantage in positive display polarity for young and old adults. Font size 12 was chosen to be readable by older patients with visual impairment. Darroch et al [17] showed that font size does not affect reading accuracy in both young and old adults, but young adults preferred size 8 and 10 while older adults preferred slightly bigger sizes of 8 to 12. Darroch et al recommend offering a setting option for font size, which was requested by two of our patients.

Button size for the questionnaire was chosen to be 20 pixels high. Anthony [18] recommends a target width of 45-57 pixels wide to allow the user’s finger to fit in the target while the edges are visible when tapping. We chose the width to be the full size of the screen, as our app needs to be comfortable for older patients with visual impairment and lower touch accuracy. After pretesting, we decided to implement a button for skipping questions, as patients can do the same in the paper-based version. Patients who do not want to (or cannot) answer one of the questions can still complete the survey.

Overall, patients were in favor of the presented app design (Figure 5); operability (95%) and navigation (93%) were rated excellent. These factors play a critical role in patient compliance, as poor user-friendliness will automatically lead to reduced usage. Completion time of the paper-based EORTC QLQ-C30 questionnaire was reported by Aaronson et al [8] as approximately 11 minutes, while our mobile version only took median 4 minutes. Both patient populations were different (median age 55 vs 63 years, international multicenter study vs in-house study) [8]. However, our study shows that patients with cancer disease are compliant with an app-based HRQoL assessment, and it is an efficient method.

To date, data are only stored locally on the device and can be transferred via a cable. In the future, patient data will be sent online, and the treating physicians will have access to the data (eg, at aftercare appointments). Here, it is crucial to guarantee data protection and security. We showed previously that 85.2% patients marked pseudonymization and data security as very important/important [10]. As health data is always highly sensitive, data security was the most crucial requirement for using an oncological app [11,19].

The first version of the app only contains the EORTC QLQ-C30 questionnaire. Future functions are planned and in progress. A multilingual approach (English, French, Italian, Russian, Arabic) is necessary, as our department is frequented by many international patients, and furthermore German is not the mother tongue of some local patients (eg, refugees). Moreover, cancer-specific modules such as the EORTC QLQ-BR23 [20] for breast cancer or QLQ-PR25 [21] for prostate cancer will be implemented next. HRQoL data from cancer patients can be used to adjust the individuals’ treatment or offer supportive therapy. Surveys on therapy satisfaction will help to improve the departments’ workflow and the patients’ contentment.

Another function of the app will be the documentation of treatment-related side effects and symptoms. The course of a disease will be monitored and chronicled. This feature can also be used to register study parameters in app-assisted randomized controlled trials (smartRCTs). SmartRCTs can reduce study duration, costs, and subject bias, as well as collect a broader range of data [11]. Certainly, a workflow regarding how to check patient answers regularly and how to handle severe entries by the patients must be developed. However, we could recently show that 94.3% of HCPs were willing to contact the patient [9], which may lead to a quicker detection of progress, as recently stated by Denis et al [22,23]. This group demonstrated that using a Web-based app resulted in a significant improvement in overall survival (12 months vs 19 months) in patients with high-risk lung cancer, and relapses were detected five weeks earlier that the control group [23].

Our usability test showed good results for the presented app. Moreover, our results demonstrate a high overall acceptance of mobile apps and telemedicine in oncology, which is in line with our previous results [9,10]. The HRQoL assessment via the app was accepted thoroughly by patients, and individuals are keen to use it in clinical routine, while data privacy and security must be ensured. Digital medicine (medicine 4.0) is an unstoppable trend and will play a significant role in the future of clinical health care.

## Appendix

Multimedia Appendix 1 QLQ-C30 questionnaire paper based version (German).

Multimedia Appendix 2 QLQ-C30 questionnaire paper based version (English).

Multimedia Appendix 3 Patient usability survey (German).

Multimedia Appendix 4 Screenshot of the review screen with completed questionnaires (administrator view).

## Abbreviations

eHealth: electronic health
EORTC: European Organization for Research and Treatment of Cancer
HCP: health care professional
HRQoL: health-related quality of life
ID: identification
mHealth: mobile health
QLQ-C30: Quality of Life Questionnaire-Core 30
QoL: quality of life
smartRCT: smart randomized controlled trial
