# Supplementary material for: Mobile App Delivery of the EORTC QLQ-C30 Questionnaire to Assess Health-Related Quality of Life in Oncological Patients: Usability Study
Source: JMIR Mhealth Uhealth. 2018 Feb 20;6(2):e45. doi: 10.2196/mhealth.9486 (PMC5840479; doi:10.2196/mhealth.9486)
Supplement: Multimedia Appendix 3 [file mhealth_v6i2e45_app3.pdf]

_ _ _

Klinikum rechts der Isar

Technische Universität München

PATIENTEN – FRAGEBOGEN

1. Wie stehen Sie dazu, dass møbile Technologien in der Medizin und auch bei uns genutzt werden (wie z.B. die App, die Sie gerade genutzt haben zu Angaben über Ihre Gesundheit)?

1	2	3	4	5	6
Finde ich gut					Finde ich nicht gut

2. Besitzen Sie privat ein Tablet oder Smartphone, das Sie für derartige Abfragen nutzen können?

□ ja □ nein

3. In der Regel füllen die Patienten solche Gesundheitsfragen in der Ambulanz auf Papier aus. Würden Sie die Durchführung mit einer mobilen App der Papierdokumentation vorziehen?

□ ja □ nein

4. Würden Sie eine derartige Anwendung auch im Alltag nutzen, um uns z.B. Ihren allgemeinen Gesundheitszustand und auftretende Nebenwirkungen während und nach der Therapie mitzuteilen?

□ ja □ nein

5. Aktuell werden Ihre Antworten lokal gespeichert und pseudonymisiert auf einen Computer zur Auswertung übertragen. Würden Sie die Daten auch pseudonymisiert über das Internet an uns senden?

□ ja □ nein

Nun zur Anwendung (App) an sich, mit der Sie eben einen Fragebogen über Ihre Gesundheit ausgefüllt haben:

6. Fanden Sie die Bedienung im Allgemeinen einfach und intuitiv?

□ ja □ nein

Was könnte man verbessern?

____

____

7. War die Navigation durch die App intuitiv? Haben Sie sich zurechtgefunden?

□ ja □ nein

Was könnte man verbessern?

____

____

Bitte wenden →

8. Wie fanden Sie das Design der App in Bezug auf…

… die Größe der Buttons (Schaltfläche)?

1	2	3	4	5	6
Finde ich gut					Finde ich nicht gut

… den Inhalt der Texte?

1	2	3	4	5	6
Finde ich gut					Finde ich nicht gut

… die Farben?

1	2	3	4	5	6
Finde ich gut					Finde ich nicht gut

… die Schriftgröße?

1	2	3	4	5	6
Finde ich gut					Finde ich nicht gut

… Inhalt pro Seite?

1	2	3	4	5	6
Finde ich gut					Finde ich nicht gut

9. Haben Sie Vorschläge, Ideen oder Kritikpunkte bezüglich der App?

____

____

10. Was hat Ihnen am besten gefallen? Was hat Sie am meisten gestört?

____

____

____

11. Haben Sie Informationen oder Funktionen vermisst?

____

____

*** Vielen Dank für die Teilnahme ***
