# Supplementary figures and images for: Mobile App Delivery of the EORTC QLQ-C30 Questionnaire to Assess Health-Related Quality of Life in Oncological Patients: Usability Study
Source: JMIR Mhealth Uhealth. 2018 Feb 20;6(2):e45. doi: 10.2196/mhealth.9486 (PMC5840479; doi:10.2196/mhealth.9486)

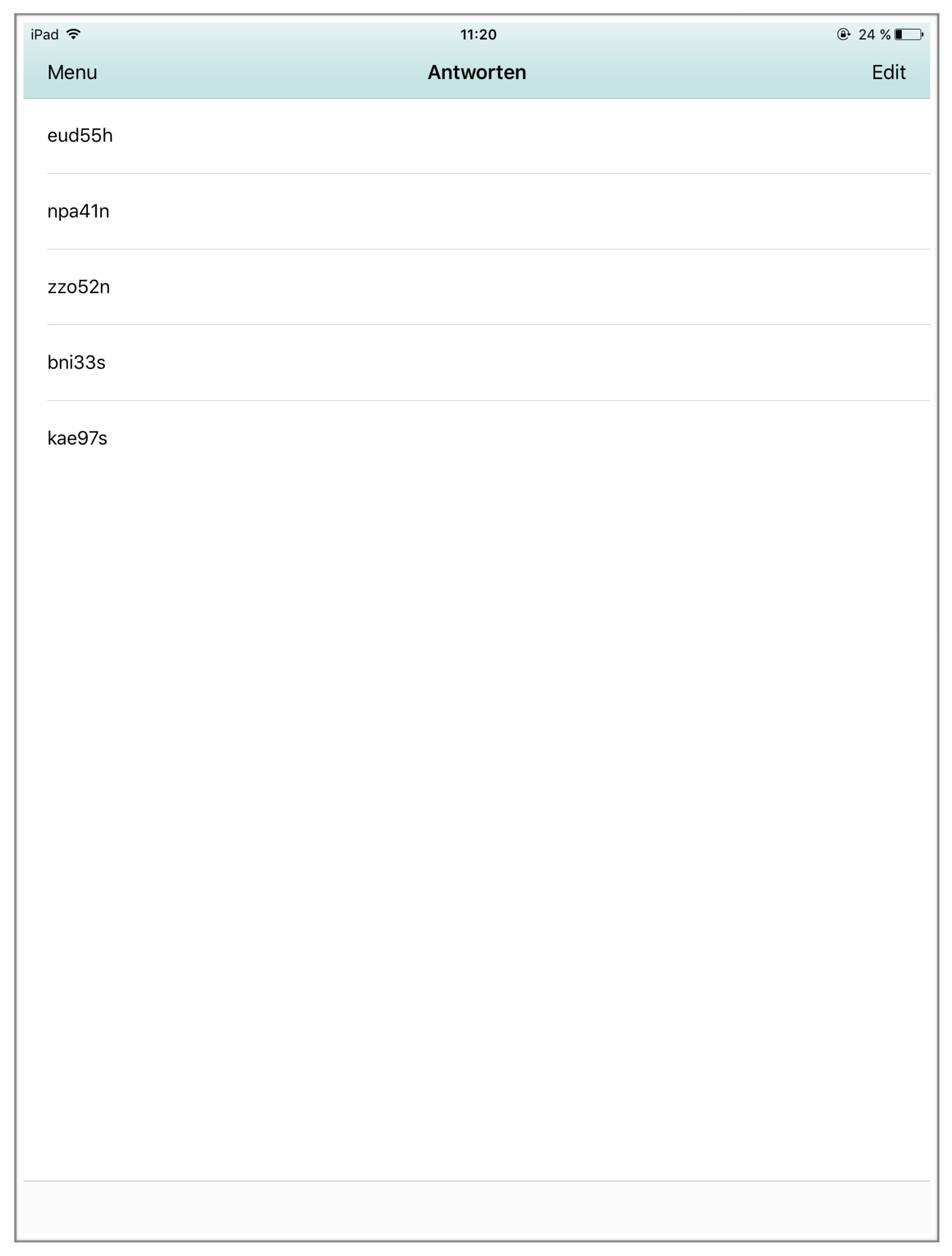

Supplement: Multimedia Appendix 4 [file mhealth_v6i2e45_app4.png]
